# Operation Mechanism of GaN-based Transistors Elucidated by Element-Specific X-ray Nanospectroscopy

**DOI:** 10.1038/s41598-018-31485-4

**Published:** 2018-09-05

**Authors:** Keiichi Omika, Yasunori Tateno, Tsuyoshi Kouchi, Tsutomu Komatani, Seiji Yaegassi, Keiichi Yui, Ken Nakata, Naoka Nagamura, Masato Kotsugi, Koji Horiba, Masaharu Oshima, Maki Suemitsu, Hirokazu Fukidome

**Affiliations:** 1 Research Institute of Electrical Communication, Tohoku University, Sendai, Japan; 2 Sumitomo Electric Industries, Ltd., Osaka, Japan; 3 Sumitomo Electric Device Innovations, Inc., Yokohama, Japan; 4 National Institute for Materials Science, Tsukuba, Japan; 5 Tokyo University of Science, Tokyo, Japan; 6 Photon Factory, High Energy Accelerator Research Organization, Tsukuba, Japan; 7 Synchrotron Radiation Research Organization, The University of Tokyo, Tokyo, Japan

## Abstract

With the rapid depletion of communication-frequency resources, mainly due to the explosive spread of information communication devices for the internet of things, GaN-based high-frequency high-power transistors (GaN-HEMTs) have attracted considerable interest as one of the key devices that can operate in the high-frequency millimeter-wave band. However, GaN-HEMT operation is destabilized by current collapse phenomena arising from surface electron trapping (SET), which has not been fully understood thus far. Here, we conduct quantitative mechanistic studies on SET in GaN-HEMTs by applying element- and site-specific photoelectron nanospectroscopy to a GaN-HEMT device under operation. Our study reveals that SET is induced by a large local electric field. Furthermore, surface passivation using a SiN thin film is demonstrated to play a dual role: electric-field weakening and giving rise to chemical interactions that suppress SET. Our findings can contribute to the realization of high-capacity wireless communication systems based on GaN-HEMTs.

## Introduction

The explosive growth of the internet of things (IoT) and *cyber-physical* systems (CPS) has dramatically increased information communication traffic^1,2^. As a result, frequency resources for information communication have significantly depleted^3^, and the exploitation of higher frequencies such as millimeter-wave frequencies has become necessary. The use of higher frequencies can enable an increase in the information transmission rate and decrease in the device power consumption per bit^4^. However, the exploitation of higher frequencies necessitates the development of high-speed information communication technologies that use high-frequency-operation transistors such as high-electron-mobility transistors (HEMTs)^5^.

A typical GaN-HEMT utilizes the two-dimensional electron gas (2DEG) formed at the GaN/AlGaN interface as the conducting channel. GaN-HEMTs are promising next-generation high-frequency devices because they provide the advantages of both high-frequency operation (due to the 2D nature of the channel electrons) and large output power due to a large bandgap^6^. In this context, GaN-HEMTs have been developed for application in high-frequency power amplifiers of satellite base stations and radar sensors. As regards their commercial use, GaN-HEMTs operating in the X-band (~10 GHz) have been commercialized by Eudyna (now acquired by Sumitomo Electric Industries)^7^. However, GaN-HEMTs are still not a mature technology since they have not fully replaced existing technologies in the millimeter-wave frequency (>60 GHz) domain.

One of the major issues that limit the performance of GaN-HEMTs is the presence of electronic traps. It has been suggested that parasitic charges moving in and out of the traps of the GaN-HEMT disperse the 2DEG density in the channel. This dispersion deteriorates the device performance by causing the so-called *current collapse phenomena*^8^. Based on correlations between the collapse phenomena and surface treatment, such as passivation with SiN thin film^9^, it has been posited that surface traps are responsible for current collapse phenomena^10^. However, the mechanism of surface electron trapping (SET) has not been fully understood thus far although SET in GaN-HEMTs has been extensively studied via electrical measurements^11^. These electrical measurements are spatially averaged and cannot be utilized to clarify the current pathway related to the trapping processes in GaN-HEMTs. As such, current knowledge on the current collapse phenomena is fairly limited.

Understanding the physical mechanisms involved in charge trapping is vital for optimizing the device performance to realize the required stable operation of GaN-HEMTs in the millimeter-wave domain. To this end, operando spectromicroscopy, i.e., spatially resolved spectroscopy of the surface electronic states during device operation, is considered an ideal tool to clarify the trapping processes. This is particularly true when this technique is combined with electrical characterization (operando characterization) because the two techniques complement each other. However, most studies on surface electronic states have been conducted using spatially averaged, non-operando spectroscopy. Some studies have utilized operando spectroscopy with a spatial resolution that is inadequate to obtain detailed insights into the device operation mechanism^12,13^. Recently, operando scanning probe microscopies with high spatial resolution, such as Kelvin probe force microscopy (KPFM), have been developed^14^. Although they provide high spatial resolution, these microscopies cannot directly probe the electronic states in an element-specific manner. This lack of element-specificity cannot ensure that the surface electronic states are truly electronically probed. For instance, KPFM without element-specificity cannot distinguish between information on the surface electronic states of the GaN-HEMT from that of carbon contaminants, which can lead to misinterpretation of the obtained results.

Against this backdrop, we have previously developed operando spectromicroscopies using soft X-ray absorption^15^ and soft X-ray photoelectron emission processes^16^, which element-specifically probe the surface electronic states. We have already demonstrated that operando X-ray photoelectron spectromicroscopy can provide information on the electronic states of a graphene transistor in an element-specific and spatially resolved manner. This was made possible by site-selectively observing the gate-bias-induced shift of the Fermi level of the channel region obtained through precise measurement of the binding energy, i.e., the energy difference between the Fermi level and the core level, which enables element-specific investigation of the Fermi level position at the solid surface^16^. By comparing the gate-bias-dependent shift with our electrical characterization results, we obtained insights into the operation mechanism of the graphene transistor, i.e., studying the interface effect with a gate insulator on the carrier modulation during operation^16^. Thus, operando photoelectron spectromicroscopy is considered adequate for clarifying the surface trapping mechanism that causes the current collapse phenomena in GaN-HEMTs.

Here, we present our operando spectromicroscopy study on a GaN-HEMT in combination with electrical characterization and device simulations. We quantitatively discuss the mechanism of surface trapping that destabilizes GaN-HEMT operation and the role of SiN surface passivation film on the GaN-HEMT surface in suppressing SET.

## Results

### Electrical evaluation of current collapse phenomena in GaN-HEMT

**Figure 1:**
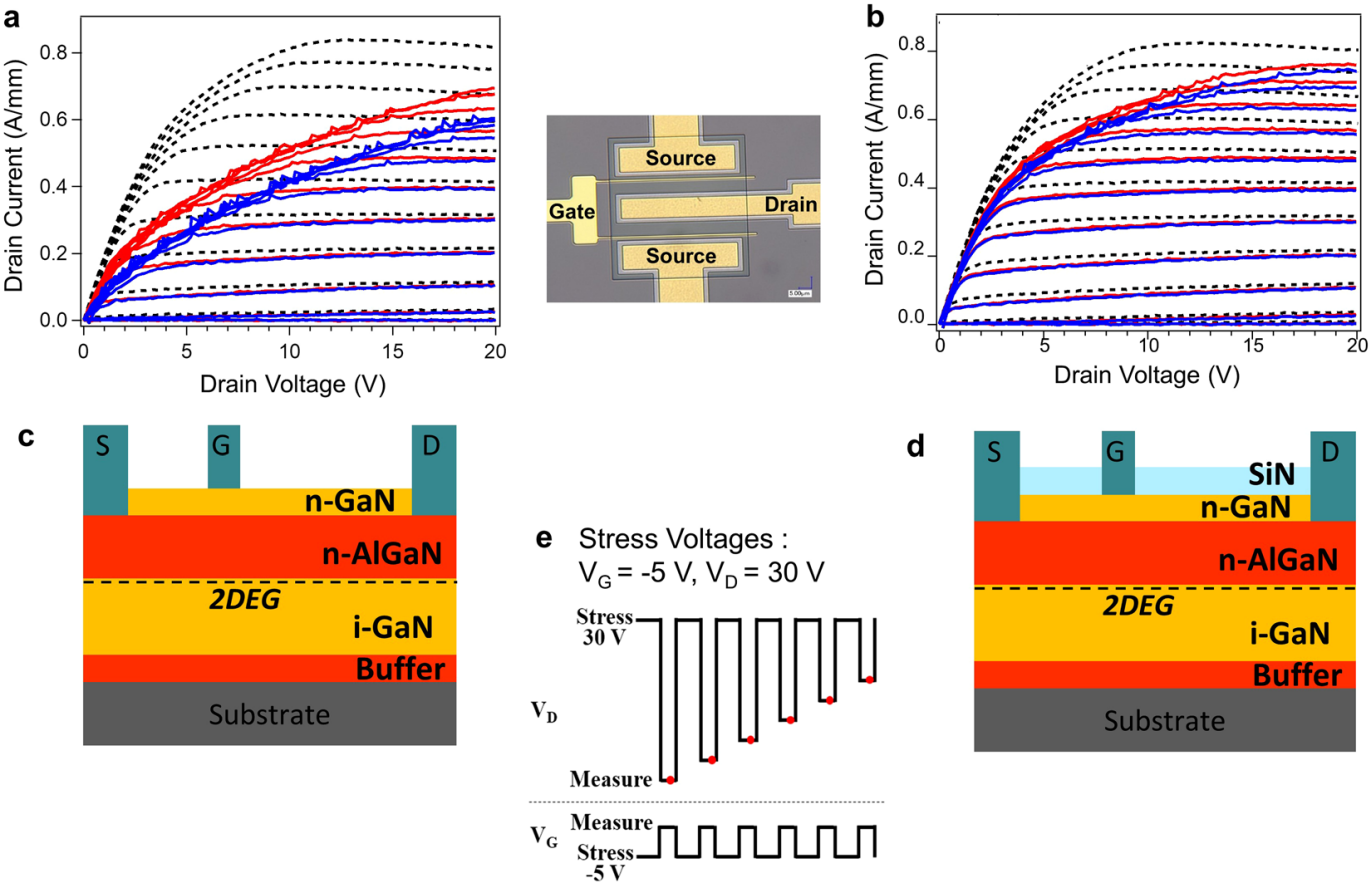
Pulsed I–V measurements of GaN-HEMT samples. (**a**,**b**) Sample without (**a**) and with (**b**) SiN thin film. The gate voltage (V_G_) is varied from −3 V to 2 V in steps of 0.5 V. The dotted line indicates the normal drain current (I_D_)–drain voltage (V_D_) characteristics. The red and blue lines denote the pulsed I_D_–V_D_ characteristics with pulse widths of 95 μs and 995 μs, respectively. The inset shows the optical micrograph of the GaN-HEMT sample. (**c**,**d)** Schematics of sample without (**c**) and with (**d**) SiN thin film. (**e**) Schematic depicting application of stress voltages during the pulsed I_D_–V_D_ measurements.

The current collapse phenomena of the GaN-HEMT samples with and without surface passivation by SiN thin film (2 nm in thickness) were electrically characterized by pulsed I–V measurements frequently used for this purpose^17^; Fig. 1 shows our I–V measurement results. In our pulsed I–V measurements, the gate and drain electrodes of the GaN-HEMT samples were subjected to short voltage pulses before each I–V measurement to ensure that the devices were in the off-state and to allow SET to occur. From Fig. 1(a,b), it is clear that the application of the stress voltage pulses substantially decreases the drain current regardless of surface SiN passivation when compared with the case of the usual DC I–V measurements. It is also clear from the comparison between Fig. 1(a,b) that the SiN thin film suppresses this drain current degradation. It is remarkable that SiN surface passivation reduces the on-resistance. Considering the charge balance between surface-trapped electrons and the 2DEG^18^, the increase in SET on the drain side near the gate electrode is related to decrease in the electron density in the channel in the drain side, thus leading to resultant increase in the on-resistance^19^. Therefore, we posit that SiN surface passivation suppresses SET on the drain side.

### Effect of negative gate bias application on surface electron trapping in GaN-HEMT

**Figure 2:**
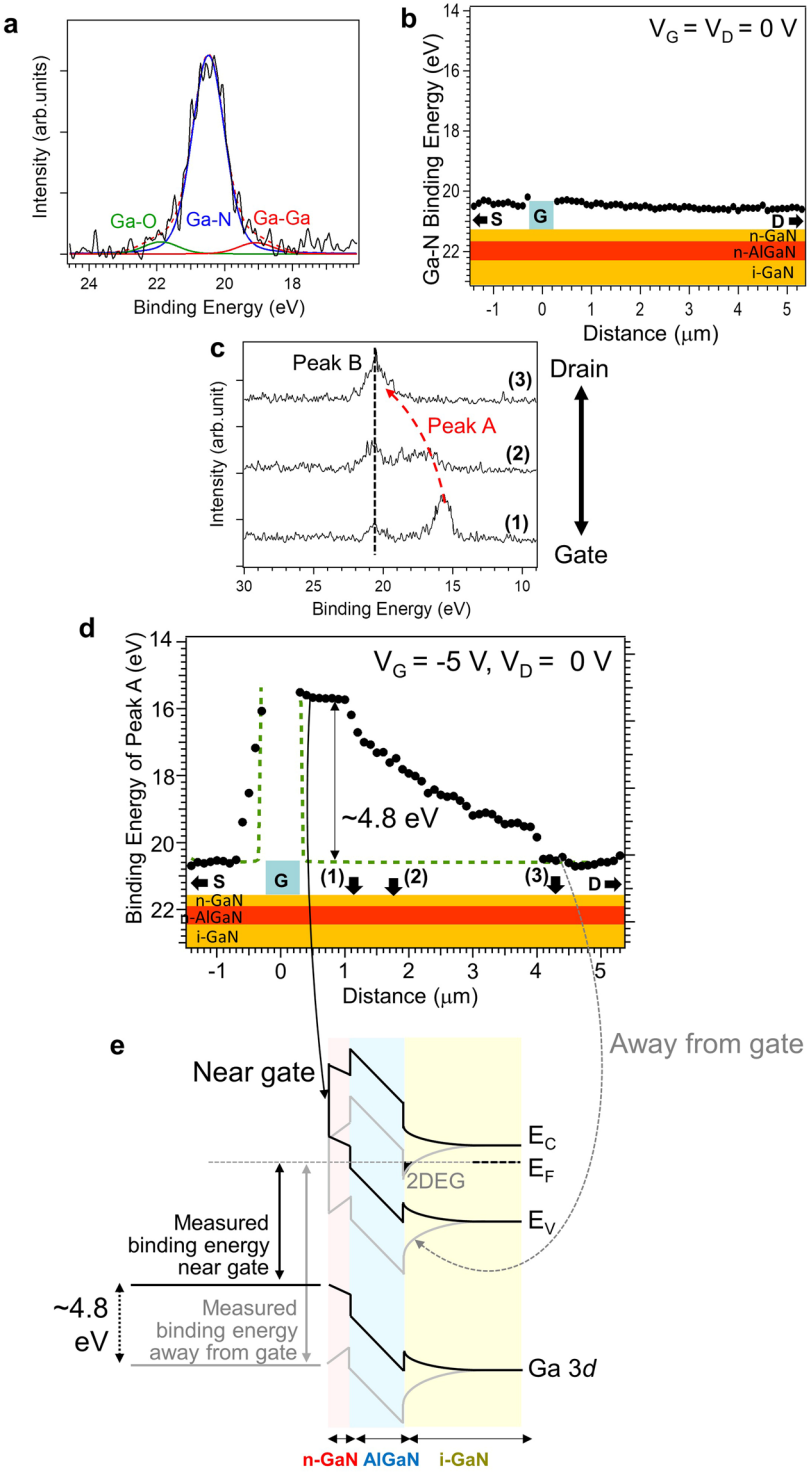
Operando photoelectron nanospectroscopy measurements of GaN-HEMT surface under gate bias application. (**a**) Typical Ga 3*d* spectrum under zero bias application. (**b**) Line profile of the binding energy of the main Ga–N peak under zero bias application. (**c**) Spatially resolved Ga 3*d* spectra under gate bias application. (**d**) Line profile of the binding energy of the main Ga–N peak under gate bias application. The green dotted line indicates the line profile in the absence of surface electron trapping. **(e)** Schematic band diagrams near the gate edge (black) and away from the gate edge (gray).

The mechanism of SET related to the current collapse phenomena cannot be clarified solely by electrical measurements. The mechanistic study of SET requires spatially resolved information about the surface electronic states because the current collapse phenomena that are suggested to be related to the local electric field formed near the gate electrode^10^ are better understood through spatially resolved information. Since the current collapse phenomena occur when a negative gate bias is applied together with a large drain bias, we first need to clarify the effect of application of the negative gate bias on the surface electronic states of the GaN-HEMT without the surface SiN thin film. For this purpose, we probed the Ga 3*d* core level using operando photoelectron nanospectroscopy; the results are shown in Fig. 2. Operando photoelectron nanospectroscopy enables the probing of SET on the topmost n-GaN layer upon setting the incident photon energy to 800 eV, corresponding to a probing depth of 2 to 3 nm^20^. Thus, the change in the potential energy of electrons at the surface of the n-GaN layer because of SET can be inferred from the spatially resolved Ga 3*d* core level spectra acquired by operando photoelectron nanospectroscopy.

Figure 2(a) shows the typical spatially resolved Ga 3*d* photoelectron spectrum without any bias application. The main spectral component arises due to the surface Ga atoms bonded with nitrogen atoms (~20.5 eV), and other tiny shoulders ascribable to Ga–O and Ga–Ga^21,22^ can also be observed. The binding energy of the main Ga–N peak exhibits a spatial independence, as can be observed from Fig. 2(b). This result indicates that the potential energy of the surface electrons does not vary spatially under zero bias application. On the other hand, the application of negative gate bias (−5 V) spatially varies the Ga 3*d* photoelectron spectra, as shown in Fig. 2(c). Near the gate edge, a new peak (peak A) appears at a lower binding energy in addition to the peak at ~20 eV (peak B), which is dominant close to the source and drain electrodes that are grounded. The difference in the binding energies of peaks A and B becomes smaller away from the gate electrode, and peak A finally merges with peak B near the drain electrode that is grounded. This change in the binding energy of peak A is related to the change in the potential energy of the electrons at the surface caused by the gate bias application. On the other hand, peak B exhibits no dependence on the gate bias, and this lack of dependence can be attributed to photoelectrons from outside the region of interest. In our X-ray focus system using Fresnel zone plate, a center stop is used to suppress the incidence of zero-order (unfocused) X-rays^20^. However, in practice, the center stop cannot completely stop the zero-order X-rays. Through the pinhole (about 80 μm in diameter) that is situated between the center stop and the sample^20^, unfocused stray X-rays can irradiate the region of non-interest (about 80 μm in diameter). Although the ratio of the unfocused stray X-rays (*I*) over the incident X-rays (*I*_0_) is quite small (*I*/*I*_0_ < 10^−3^), the integrated intensity from the region of “non-interest” due to the unfocused stray X-rays can be comparable with that from the region of interest due to the presence of focused X-rays. Peak B, which did not show a significant shift in binding energy, is thus assignable to photoelectrons from regions where no bias application was applied because of the unfocused stray x-rays. The microscopic information on the surface electronic states will hereafter be analyzed using peak A.

The line profile of the binding energy of peak A confirms that SET occurs near the gate edge under gate bias application. If SET is negligible, the applied negative potential at the gate should drop within 100 nm from its edge, as schematically indicated by the green dotted line in Fig. 2(d). However, the binding energy or potential energy of the electrons at the surface actually changes quite gradually away from the gate edge. This result is ascribable to SET near the gate edge, as schematically shown in Fig. 2(e). This scenario can be described as follows. Near the drain and source electrodes, SET does not occur because the electrodes are far away from the gate edge under bias. In this case, the bands bend downward in the n-GaN thin film, and the 2DEG is present at the AlGaN/i-GaN interface, as schematically indicated by the gray line in Fig. 2(e). In contrast, SET occurs in the regions near the gate edge. Correspondingly, the 2DEG is absent at the AlGaN/i-GaN interface because of the equilibration between the surface-trapped electrons and the 2DEG^18^, and the bands bend upward in the n-GaN thin film. This changes the potential that electrons experience at the surface, thereby leading to lowering of the binding energy of the Ga 3*d* electrons in this region. Assuming that the energy difference between the binding energies near the gate edge and the drain electrode (4.8 eV, as shown in Fig. 2(d)) is caused by SET, the density of the surface-trapped electrons near the gate edge can be roughly estimated by means of the following equation:

1 $${N}_{trap}=\frac{{\varepsilon }_{0}{\varepsilon }_{GaN}{\varepsilon }_{AlGaN}}{q({d}_{GaN}{\varepsilon }_{AlGaN}+{d}_{AlGaN}{\varepsilon }_{GaN})}\cdot {\rm{\Delta }}{V}_{BE},$$

In the above equation, *ΔV*_*BE*_ represents the binding energy difference (4.8 eV) near the gate edge, *d*_*GaN*_, *d*_*AlGaN*_ the thicknesses of the surface n-GaN layer and AlGaN layer beneath the surface n-GaN layer, respectively, and *ε*_0_ and *ε*_*GaN*_ and *ε*_*AlGaN*_ the dielectric constant in vacuum and the relative dielectric constants of n-GaN and AlGaN, respectively. As per our calculations, *N*_*trap*_ = 9.5 × 10^12^ cm^−2^; this value is plausible because it is similar to that reported in a previous work based on macroscopic electrical measurements^23^. Thus, our results demonstrate clearly that SET occurs near the gate edge under the application of a negative gate bias.

### Effect of application of both gate and drain voltages on surface electron trapping

**Figure 3:**
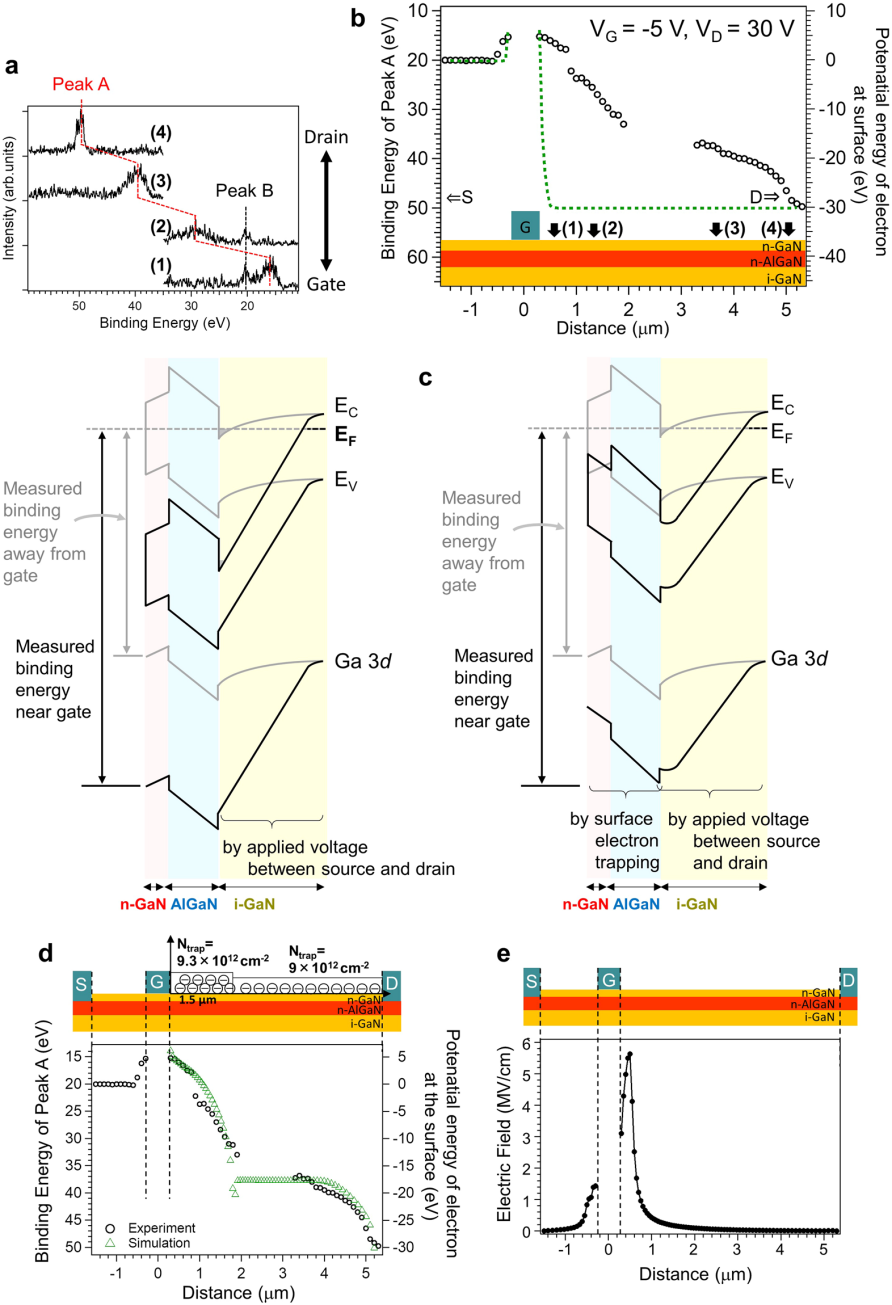
Operando photoelectron nanospectroscopy results of GaN-HEMT surface under application of both drain and gate biases. (**a**) Typical Ga 3*d* spectra under application of drain (30 V) and gate (−5 V) biases. (**b**) Line profile of the binding energy of the main Ga–N peak under application of drain (30 V) and gate (−5 V) biases. **(c**) Schematic band diagrams corresponding to (left) absence of surface electron trapping, and (right) occurrence of surface electron trapping. **(d**) Simulations utilized to reproduce the experimental data by assuming surface electron densities. (**e**) Estimated local electric field distribution under application of gate (−5 V) and drain (30 V) biases.

In the previous section, we demonstrated the occurrence of SET under negative gate bias application. In the actual electrical measurements described earlier, the current collapse phenomena are observed under application of not only a negative gate voltage but also a positive drain voltage. Thus, to evaluate current collapse under actual operating conditions, we utilized operando photoelectron nanospectroscopy in combination with device simulation to study the case where both the gate and drain voltages are applied to the device. Figure 3(a) displays the typical spatially resolved Ga 3*d* spectra under application of both the drain (30 V) and gate (−5 V) voltages. Similar to the result obtained under application of only the gate voltage, we observe a remarkable position dependence of the main Ga 3*d* peak (peak A) in the region of interest. There also appears a peak B that shows no position dependence. The application of the drain voltage (30 V) shifts the point of zero potential away from the drain electrode. Therefore, peak B from the region of zero potential appears in the spatially-resolved spectra acquired away from the drain electrode, in contrast to the case in Fig. 2(c) wherein the drain voltage is zero. Figure 3(b) shows the line profile of the binding energy of peak A under gate and drain voltage stresses. In the region corresponding to a distance of 2 to 3 μm from the gate, the Ga 3*d* peak position is not plotted because the spectra were too weak for us to determine the peak position precisely. It is clear that the binding energy drops more gradually near the gate edge, and its gradient is less than that of the curve obtained under only gate voltage application. Qualitatively, this change in the binding energy can be explained by considering the band diagram shown in Fig. 3(c). If SET does not occur, the density of the 2DEG in the observed region does not change. The band structures of the AlGaN/i-GaN interface, n-GaN, and AlGaN layers between the gate edges and source or drain electrodes also show no changes. The voltage applied between the source and drain electrodes changes the potential in the bulk of the i-GaN layer. On the other hand, if SET occurs, the 2DEG density is depleted accordingly. The band structure of n-GaN and that at the AlGaN/i-GaN interface are changed by SET. In the i-GaN layer, the band structure is determined mainly by the voltage applied between source and drain electrodes.

For a quantitative analysis of SET, we analyzed the binding energy reflecting the potential energy of electrons at the surface by using a device simulator, which is frequently used for studying the device characteristics of GaN-HEMTs^24^. This simulation was performed for the surface region between the gate and drain electrodes, which was previously speculated to affect the on-resistance. In this simulation, we estimated the conduction band energy at the surface, which is equivalent to the potential energy of the electrons at the surface. As can be observed in Fig. 3(d), the simulations quantitatively reproduce the experimental data upon assumption of a two-step surface electron density distribution in the drain side. This simulation result demonstrates that SET occurs not only near the gate edge but also away from the gate edge. The density of the surface-trapped electrons just near the gate edge (9.3 × 10^12^ cm^−2^) is larger than that away from the gate edge (9.0 × 10^12^ cm^−2^). A possible reason for this is that a large local electric field is formed near the gate edge^25^. In fact, the simulation result indicates the existence of a large local electric field near the gate edge (Fig. 3(e)). Although the large local electric field explains SET near the gate edge, it cannot explain the substantial trapping in the second region away from the gate edge where the local electric field is relatively small. It is possible that the conduction of trapped electrons via trap-to-trap hopping occurs with assistance from the local electric field in the drain side^26^, which may play a role in trapped-electron generation away from the gate electrode. The estimated SET density is consistent with that roughly estimated from the line profile under gate bias application. SET in the drain side reduces the electron density in the drain side of the channel because of the equilibration between the density of the surface-trapped electrons and the 2DEG density^18^. The reduced 2DEG density in the drain side increases the resistance of the drain side of the channel, thus resulting in increase in the on-resistance. This explains the large on-resistance observed in the I–V characteristics of the GaN-HEMT without SiN passivation (Fig. 1(a)). Thus, surface trapping under gate and drain voltage application with regard to current collapse phenomena is quantitatively characterized by operando photoelectron nanospectroscopy with element- and site-specificity that cannot be realized by other characterization methods such as I–V measurements and scanning probe microscopy (SPM).

### Role of SiN surface passivation

**Figure 4:**
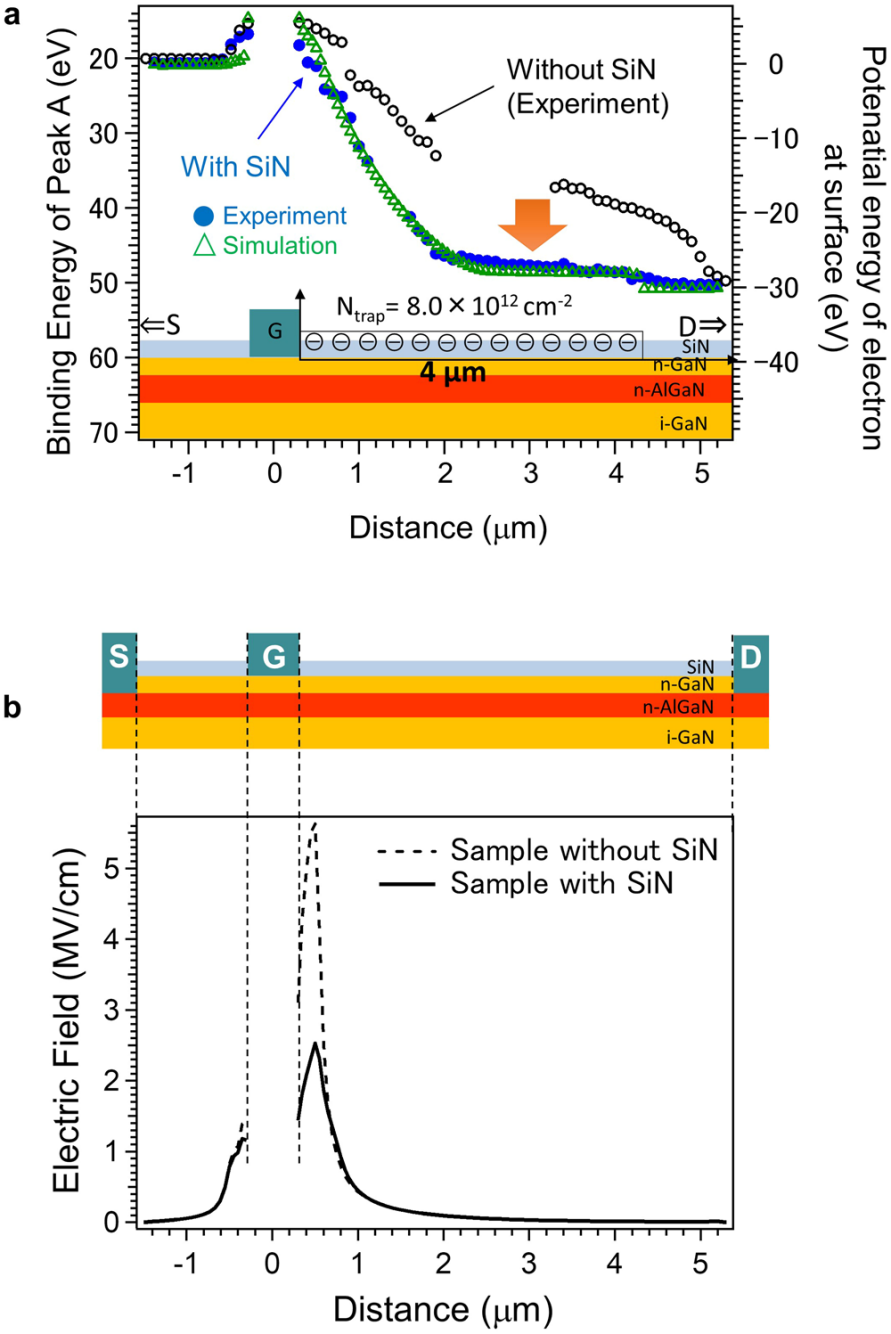
Operando photoelectron nanospectroscopy results utilized to clarify effect of SiN passivation. (**a)** Line profile of the binding energy of the main Ga–N peak of the GaN-HEMT with SiN surface passivation under application of drain (30 V) and gate (−5 V) biases. (**b**) Estimated local electric field distribution with (thick line) and without (dotted line) SiN surface passivation.

Surface passivation via deposition of SiN thin films on the surface of the GaN-HEMT suppresses the current collapse^9^. Such suppression in the presence of the SiN passivation film has been ascribed to reduction in the surface states^10,14^. Reduction by SiN passivation may be related to reduction in nitrogen-related vacancies^21^ or that of oxygen bonded with surface Ga atoms^27^. However, the underlying details of the mechanism of SiN surface passivation (for instance, the region where the surface state density is reduced) have not been fully understood thus far. Our operando photoelectron nanospectroscopy results also clarify the role of the SiN surface passivation (2 nm in thickness). The macroscopic pulsed I–V measurements (Fig. 1) reveal

that surface passivation by the SiN thin film reduces the on-resistance. This reduced on-resistance is speculated to originate from the decrease in the density of surface-trapped electrons in the drain side because of the consequential increase in the 2DEG density in the drain side. To microscopically clarify how SiN surface passivation affects the surface electronic states, we measured the spatially resolved Ga 3*d* spectra under gate and drain voltage stresses (Fig. 4(a)). The binding energy of the Ga 3*d* peak drops rapidly near the gate edge when compared with the case without SiN passivation. Here, we note that spatially resolved spectra can be acquired precisely regardless of the presence of the SiN thin film. We speculate that the SiN thin film may induce the build-up of positive charges (core holes) at the surface, which are formed due to photoelectron emission. However, positive charges are easily compensated (or neutralized) by the source and metal electrodes because the electrodes are located in the vicinity of the observed region, and further, the SiN thin film is too thin to prevent this neutralization. In addition, operando observations with the use of the same instrument as in this study have already demonstrated that the energy position of the Fermi level for a graphene transistor with graphene deposited on a SiO_2_ thin film as a channel under gate voltage application can be precisely determined^16^. This result clearly indicates that SiN surface passivation reduces the amount of SET. To quantify the effect of SiN passivation on the surface electronic states, we again analyzed the line profile of the binding energy of the Ga 3 peak by using the device simulator (green triangles). The observed remarkable change in the line profile is attributable to decrease in surface-trapped electrons in addition to the presence of the SiN dielectric thin film that reduces the potential energy of the electrons at the surface via dielectric screening. Our analysis resulted in the following findings: First, the assumption of a single-step distribution of the density of the surface-trapped electrons can account for the complete device behavior; the density of the surface-trapped electrons just near the gate edge is the same as that away from the gate edge. This is in sharp contrast with the case of the GaN-HEMT without SiN passivation, wherein the density of the surface-trapped electrons is larger just near the gate edge than that away from the gate edge. The device simulator results suggest that the local electric field near the gate edge is weakened drastically by the presence of the SiN thin film (Fig. 4(b)). This drastic weakening of the local electric field may account for the disappearance of the larger density of surface-trapped electrons just near the gate edge. Second, SiN passivation reduces the estimated density of the surface-trapped electrons by about 10% both near and away from the gate edge. This site-independent reduction in the density of the surface-trapped electrons cannot be explained solely by the weakened local electric field because the local electric field changes mostly near the gate edge and scarcely changes in the region away from the gate edge. A possible explanation is that the SiN thin film chemically interacts with the GaN-HEMT surface by forming chemical bonds, which decreases the local density of the surface traps^29^. Thus, we conclude that the role of SiN passivation in GaN-HEMT is two-fold: weakening of the local electric field and reduction in the number of surface-electron traps.

## Conclusion

Operando photoelectron nanospectroscopy is a powerful tool to clarify surface-related phenomena that influence electronic device performance because of its ability to probe the surface electronic states in an element- and site-specific manner. By combining this method with I–V measurements mediated by device simulation, we succeeded in quantitatively clarifying the mechanism of surface electron trapping responsible for the current collapse phenomena in GaN-HEMTs. Our work can contribute to the stable operation of GaN-HEMTs at higher frequencies for future “smart society” applications. Here, we note that operando photoelectron nanospectroscopy is applicable to most semiconductor materials and devices. Our future work will focus on considering the time resolution of operando photoelectron nanospectroscopy in addition to spatial resolution. Some of the authors of this study are exploiting spatiotemporal operando spectromicroscopy by using pulsed X-rays, and their results will be presented elsewhere.

## Methods

### Sample preparation

The GaN-HEMT samples used in the study were supplied by Sumitomo Electric Industries and Sumitomo Electric Device Innovations. The samples were fabricated by growing an i-GaN layer on SiC substrates, followed by epitaxial growth of n-AlGaN and n-GaN layers, and final fabrication of the source, drain, and gate electrodes. A SiN passivation film was deposited on the n-GaN surface. The thickness of the SiN passivation film was 2 nm, which is sufficiently thin to allow probing of the GaN-HEMT surface.

### Operando Photoelectron nanospectroscopy

The spatially resolved Ga 3*d* core-level photoelectron spectra were acquired by the use of the 3D nano-ESCA system installed at the BL07LSU at SPring-8^22^, where the synchrotron radiation (SR) beam has a high energy-resolving power (*E*/Δ*E* > 10^4^). The photon energy of the SR beam for the measurement was 800 eV. The use of 3D nano-ESCA enabled us to obtain a high lateral resolution (70 nm) by focusing X-rays using a Fresnel zone plate. The energy resolution of the spectrometer was set to 0.3 eV, and the accuracy of the angle resolution was 0.9°. The binding energy scale was calibrated by the photoelectron peaks of a gold foil (Au 4 *f*_7/2_). The samples were mounted on a sample holder enabling electrical connections to the source, drain, and gate electrodes.

### Device simulation

The device simulator ATLAS (SILVACO Inc.) was used for the simulation of the conduction energy, equivalent to the potential energy of electrons, and the local electric field distribution formed by the applied biases. ATLAS simulations have been proved as being adequate to analyze GaN-HEMT operation. In the simulations in this study, the charge densities for n-AlGaN/i-GaN and n-GaN/n-AlGaN interfaces were set to be +8.5 × 10^12^ cm^−2^ and −8.5 × 10^12^ cm^−2^, respectively, to reproduce the I–V characteristics of the GaN-HEMT.
